# COPD Imaging on a 3rd Generation Dual-Source CT: Acquisition of Paired Inspiratory-Expiratory Chest Scans at an Overall Reduced Radiation Risk

**DOI:** 10.3390/diagnostics10121106

**Published:** 2020-12-18

**Authors:** Joshua Gawlitza, Thomas Henzler, Frederik Trinkmann, Elke Nekolla, Holger Haubenreisser, Gunnar Brix

**Affiliations:** 1 Clinic of Diagnostic and Interventional Radiology, Saarland University Medical Center, 66424 Homburg, Germany; 2 Institute of Clinical Radiology and Nuclear Medicine, University Medical Center Mannheim, 68159 Mannheim, Germany; thomas.henzler@me.com; 3 Pulmonology and Critical Care Medicine, Thoraxklinik at University Hospital Heidelberg, Translational Lung Research Center Heidelberg (TLRC), German Center for Lung Research (DZL), 69115 Heidelberg, Germany; frederik.trinkmann@med.uni-heidelberg.de; 4 Department of Biomedical Informatics of the Heinrich-Lanz-Center, University Medical Center Mannheim, Medical Faculty Mannheim, Heidelberg University, 69115 Heidelberg, Germany; 5 Department of Medical and Occupational Radiation Protection, Federal Office for Radiation Protection, 91465 Neuherberg, Germany; enekolla@bfs.de (E.N.); gbrix@bfs.de (G.B.); 6 Radiological Joint Practice Ludwigsburg, 17509 Ludwigsburg, Germany; holger.haubenreisser@gmail.com

**Keywords:** COPD, computed tomography, radiation dose, radiation risk

## Abstract

As stated by the Fleischner Society, an additional computed tomography (CT) scan in expiration is beneficial in patients with chronic obstructive pulmonary disease (COPD). It was thus the aim of this study to evaluate the radiation risk of a state-of-the-art paired inspiratory-expiratory chest scan compared to inspiration-only examinations. Radiation doses to 28 organs were determined for 824 COPD patients undergoing routine chest examinations at three different CT systems–a conventional multi-slice CT (MSCT), a 2nd generation (2nd-DSCT), and 3rd generation dual-source CT (3rd-DSCT). Patients examined at the 3rd-DSCT received a paired inspiratory-expiratory scan. Organ doses, effective doses, and lifetime attributable cancer risks (LAR) were calculated. All organ and effective doses were significantly lower for the paired inspiratory-expiratory protocol (effective doses: 4.3 ± 1.5 mSv (MSCT), 3.0 ± 1.2 mSv (2nd-DSCT), and 2.0 ± 0.8 mSv (3rd-DSCT)). Accordingly, LAR was lowest for the paired protocol with an estimate of 0.025 % and 0.013% for female and male patients (50 years) respectively. Image quality was not compromised. Paired inspiratory-expiratory scans can be acquired on 3rd-DSCT systems at substantially lower dose and risk levels when compared to inspiration-only scans at conventional CT systems, offering promising prospects for improved COPD diagnosis.

## 1. Introduction

With an incidence of over 300 million persons and 3.17 million deaths per year worldwide, chronic obstructive pulmonary disease (COPD) is a frequent and severe inflammatory disorder of the lungs [1,2]. Computed tomography (CT) is increasingly used in these patients to quantify the amount of emphysema and small airway involvement [3,4]. In contrast to other pulmonary entities, an additional expiratory scan has been described to offer beneficial information in patients with COPD as it better identifies areas of air trapping which is a surrogate of small airway inflammation in patients with COPD [3,5,6]. Not only do expiratory CT scans show a stronger correlation with airway and lung function parameters, but they also allow for the quantification of air trapping and residual volume [6,7]. This additional value of an expiratory CT scan was underlined in a statement by the Fleischner Society in 2015 [8]. However, a paired inspiratory-expiratory CT examination unavoidably increases the radiation exposure and risk of COPD patients compared to an inspiration-only examination performed at the same CT system. Thus, this issue has to be addressed carefully.

On the other hand, several hardware and software innovations—such as automated tube current modulation, low voltage imaging, spectral shaping, dynamic collimation, as well as iterative image reconstruction—have substantially decreased the radiation dose of CT scans [9,10,11,12,13,14,15]. These hardware and software techniques are meanwhile available in clinical routine by using high-end CT systems and may help to (over-)compensate the increased radiation exposure associated with the acquisition of paired inspiratory-expiratory scans of COPD patients.

It was thus the aim of the present retrospective study to investigate this concept from a radiation-hygienic perspective based on a large cohort of COPD patients who underwent chest CT examinations at three different CT systems—a conventional multi-slice CT (MSCT), a dual-source CT of the 2nd generation (2nd-DSCT), and a dual-source CT of the 3rd generation (3rd-DSCT). Based on the equivalent organ doses determined for these patients, effective doses as well as age- and sex-specific lifetime attributable cancer risks (LAR) were estimated.

## 2. Methods

### 2.1. Subjects, CT Systems, and Imaging Protocols

The study design was in accordance with the Declaration of Helsinki and was approved by the local ethics committee (approval number 2015-415M-MA, dated at 14 April 2015) of our institution (blinded for review). The study was registered at clinicaltrials.gov (blinded for review).

We retrospectively analyzed CT scans of COPD patients performed over a three-year period in regular health care at three different CT systems of the same manufacturer (Siemens Healthineers, Forchheim, Germany): a conventional MSCT (Somatom Emotion 16), a 2nd-DSCT (Somatom Definition FLASH), and a 3rd-DSCT (Somatom FORCE) equipped with a tin filter for spectral shaping [13,14,16]. Relevant scanner characteristics and parameter settings are summarized in Table 1. Chest imaging was performed at all systems in inspiration, immediately followed by an additional expiration scan in case of patients examined at the 3rd-DSCT. The thickness and matrix size of the reconstructed CT images were identical for all scanners and thus enable a comparative assessment of the image quality. Excluded from the study were patient examinations with a scan length above 36 cm that is atypical for COPD imaging.

### 2.2. Dosimetry

The average equivalent doses of 28 tissues and organs defined in International Commission on Radiological Protection (ICRP) publication 103 [16] were estimated for each individual CT scan from the respective dose-length product (DLP) by using scanner- and sex-specific dose conversion coefficients. These coefficients were determined by means of a dedicated CT dosimetry software (CT-Expo; Hamburg/Hannover, Germany [17]; for evaluation see [18]) for both a female and male reference person for chest scans ranging from cervical vertebra 4 to about 3 cm below the diaphragm. The dose conversion coefficient for the effective dose was computed from the organ coefficients by using the tissue weighting factors given in ICRP publication 103. As a robust and scientifically sound metric to assess the comparability of patient groups regarding the transaxial attenuation of X-rays in the thorax region, the water-equivalent diameter (WED) was computed [19,20].

### 2.3. Assessment of Image Quality

To compare the quality of chest scans acquired in inspiration at the three CT systems, the signal-to-noise ratio (SNR) of lung tissue was determined for mixed-sex subgroups of patients, each comprising 18 patients. To this end, mean CT densities and the respective standard deviations were computed for regions-of-interest (ROIs; area, 2 cm^2^) placed over the left lung segment 3. In case of nodules, dystelectasis, infiltrations, or other pulmonal findings in this lung segment, ROIs were placed over the left segment 1. The variation of CT densities in the ROIs depends on various factors—among others quantum noise, the various data processing steps, the reconstruction algorithm, and the microstructure (texture) of lung tissue—and is thus an appropriate parameter for comparing chest CT images acquired under various conditions.

### 2.4. Lifetime Attributable Cancer Risk

The lifetime attributable risk (LAR) for cancer incidence from major tumor sites, i.e., the excess lifetime risk of developing cancer associated with the radiation, was calculated from the gender-specific organ doses by using the organ-, sex-, and age-specific risk models developed by the BEIR (National Academy of Sciences Advisory Committee on the Biological Effects of Ionizing Radiation) VII committee [21], assuming a linear non-threshold dose-response relationship. Following the precautionary principle in medical radiation protection, and complying with the recommendation of the German Commission on Radiological Protection [22], a dose and dose-rate effectiveness factor of 1 was applied. Risk estimates were adapted to the German general population. Details of the risk estimation are described in a previous publication [23].

### 2.5. Data Analysis

The central tendency and variability of data were described by the arithmetic mean and the standard deviation (MW ± SD), the distribution of data was presented by box-and-whisker plots. Statistical analysis was performed using JMP 12 (SAS; Cary, NC, USA). A two-sided Kruskal–Wallis ANOVA with post-hoc Dunn’s testing was used to test for significant differences between different patient groups or CT protocols. A *p*-value below 0.05 was considered as significant.

## 3. Results

In total, 824 COPD patients were included into the study. Their allocation to the six patient groups (three scanners/protocols, 2 sexes) as well as their age, WED, and scan length are summarized in Table 2. For both sexes, the differences between the patient groups examined at the three CT systems were statistically significant, although they are relatively small (age: <10%, WED: <13%, scan length: <8%). The severity and burden of COPD varied between individuals, but in any case, GOLD (global initiative of chronic obstructive lung disease) stages were equal to or greater than 2 for all patients.

**Figure 1:**
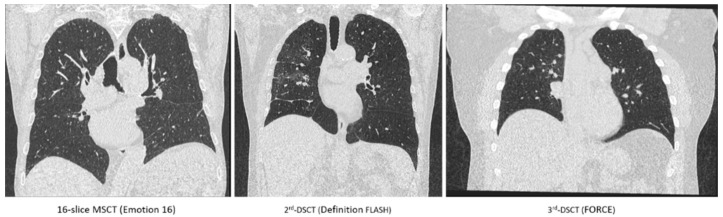
Representative coronal CT sections of the lung reconstructed from data acquired in inspiration for three different patients at the CT systems considered using the scan protocols summarized in Table 1 (identical windowing of Hounsfield values).

**Figure 2:**
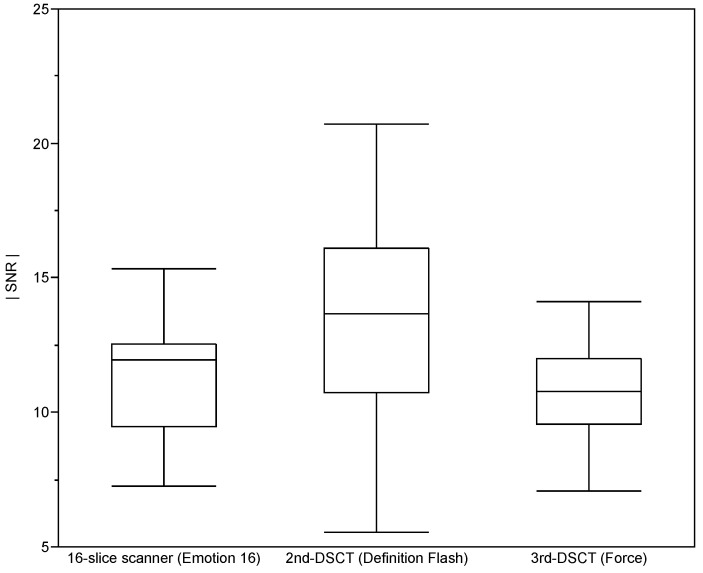
Signal-to-noise ratios for a representative lung region evaluated from CT scans acquired at the three CT systems in inspiration using the three protocols summarized in Table 1. Box-and-Whiskers-Plot representation after Tukey [24].

Figure 1 shows representative chest images acquired at the three considered CT systems. Differences in the signal-to-noise ratios of lung tissue determined for the three systems/protocols were not significant (*p* = 0.098) as seen in Figure 2.

**Figure 3:**
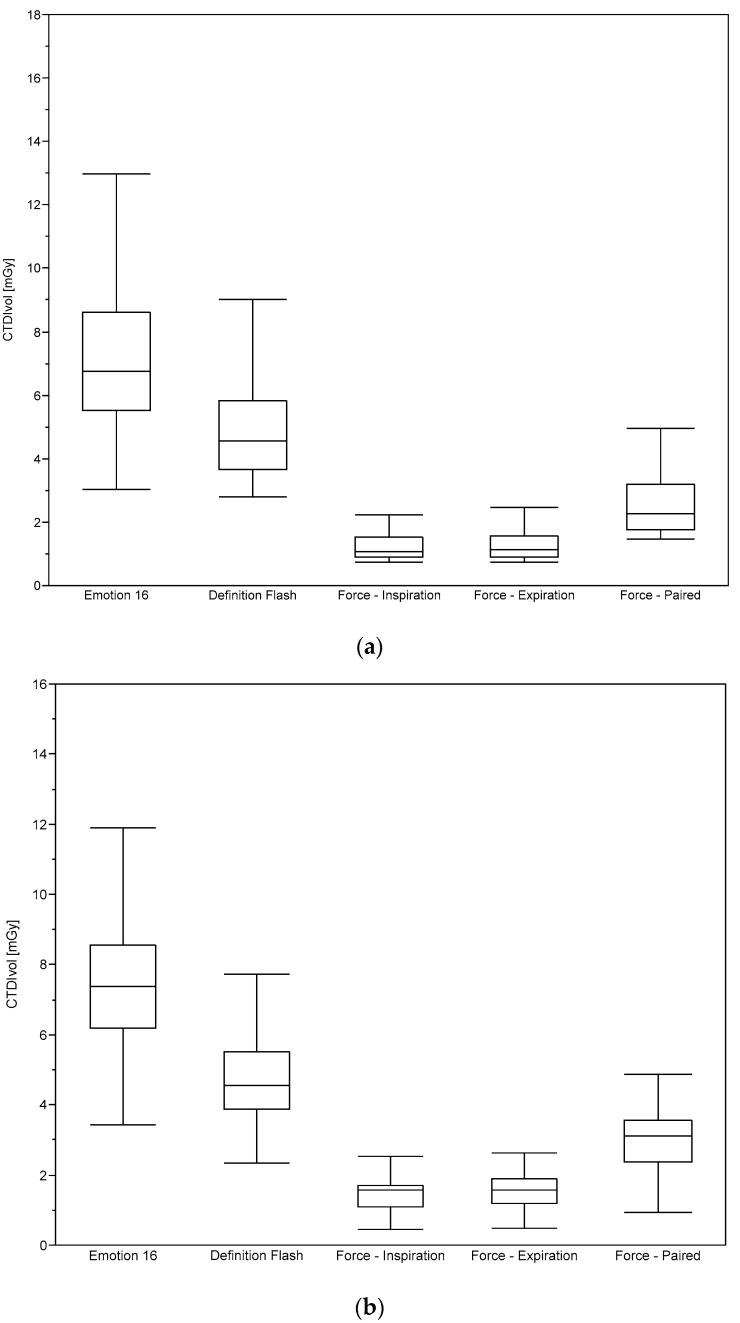
Volume CT dose index (CTDI_vol_) evaluated for (**a**) female and (**b**) male patients undergoing chest scans using the five protocols considered.

Volume CT dose indices (CTDI_vol_) determined for female and male patients undergoing chest scans at the three considered CT systems are presented in Figure 3a,b as box-and-whisker-plots. The CTDI_vol_ values decrease considerably with the increasing technical performance of the systems. The figures reveal highly significant differences between the five investigated protocols for both female and male COPD patients. 

**Figure 4:**
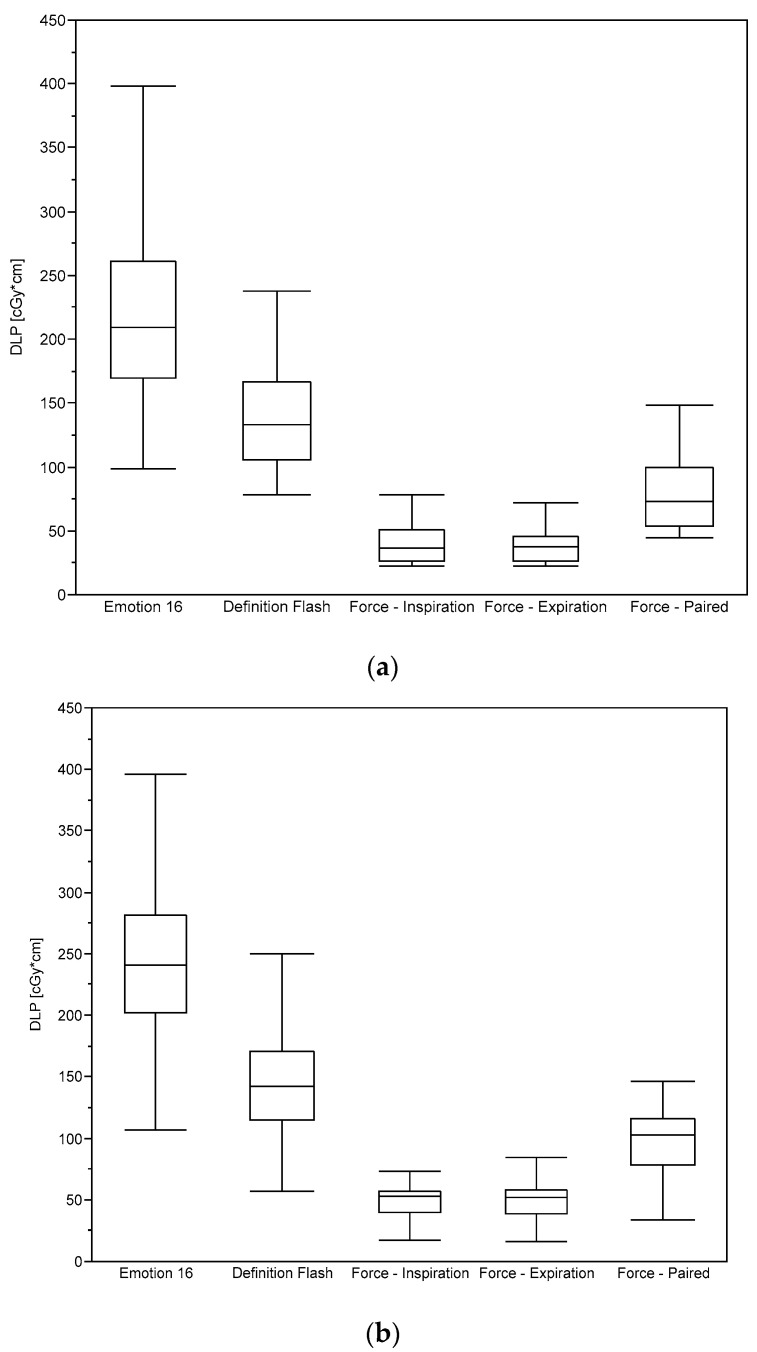
Dose-length-product (DLP) evaluated for (**a**) female and (**b**) male patients undergoing chest scans using the five protocols considered.

As shown in Figure 4a,b, essentially the same holds for the distribution of the dose-length products (DLP). Taking into account that the scan length does not differ markedly between the patient groups (cf. Table 2), this is in line with the fact that the DLP is defined as the product of the CTDI_vol_ and the scan length. 

**Figure 5:**
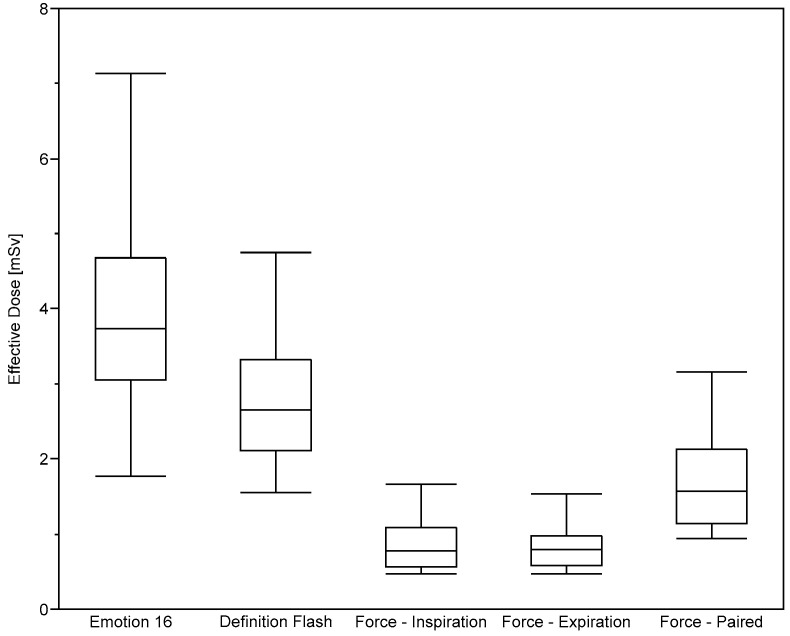
Effective dose evaluated for patients undergoing chest scans using the five protocols considered.

In accordance with these results, lowest effective doses were found for the 3rd-DSCT as shown in Figure 5. Equivalent doses for the ten most-highly exposed organs are given in Table 3 for female and male patients undergoing chest CT scans at the three CT systems. As expected, the highest values occurred for the organs lying completely or partially in the useful beam. Organ doses are highest at the MSCT system and lowest at the 3rd-DSCT. For the lungs exposed in inspiration, for example, equivalent doses are reduced for females/males by 29/24% at the 2nd-DSCT and by 77/76% at the 3rd-DSCT compared to the MSCT. For the same breathing phase, lung doses at the 3rd-DSCT are reduced by about 70/67% compared to the 2nd-DSCT.

**Figure 6:**
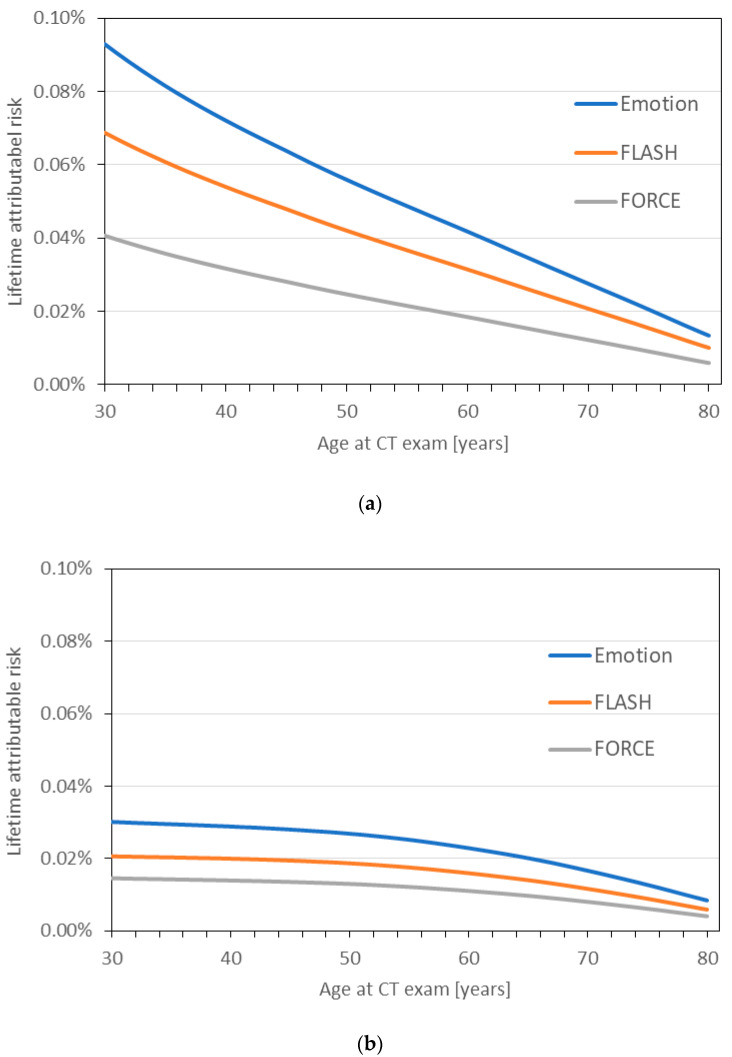
Lifetime attributable cancer risk (LAR) for (**a**) female and (**b**) male reference patient undergoing either inspiration-only chest scans at the MSCT and 2nd-DSCT or a paired inspiratory-expiratory scan at the 3rd-DSCT (incidence data).

Sex-specific LAR curves are shown in Figure 6a,b in dependence on age at exposure. Overall, LAR was higher for female patients and decreases considerably for both sexes with increasing age at CT. For the paired inspiratory-expiratory protocol at the 3rd-DSCT, for example, the LAR varies from 0.04% to 0.006% in the age interval of 30 to 80 years at CT exposure for female patients, and from 0.015% to 0.004% for male patients. LAR values estimated for this protocol are only about half as high as for the inspiration-only protocol at the MSCT.

## 4. Discussion

The aim of the present study was to evaluate the feasibility to acquire paired inspiratory-expiratory chest scans at a 3rd-DSCT without increasing the radiation risk compared to inspiration-only scans performed at a MSCT or 2nd-DSCT that are still widely used in clinical practice. To this end, radiation exposures and risks were investigated for three groups of COPD patients undergoing chest examination at these CT systems.

Besides the technological features of the CT systems, radiation exposure of patients is considerably affected by their stature. Therefore, the patient groups should be comparable as far as possible regarding the WED and the scan length. Due to the large number of patients, a Kruskal–Wallis test yielded for both parameters significant differences for the patients examined at the three scanner types. However, the mean values of the WED and the scan length determined for the three groups differed by less than 13% and 8%, respectively. From a pragmatic point of view, this is a sufficiently well match of the patient groups so that a substantial body-size related bias in the dose estimates can reasonably be excluded [20]. Moreover, the mean WED was highest for the group of patients scanned at the 3rd-DSCT which tends to underestimate the dose differences to the two other systems. The small differences in the WED between the patient groups thus strengthen even more the evidence of our finding that paired inspiratory-expiratory scans at a 3rd-DSCT can be performed at a substantially lower radiation dose and smaller risk compared to inspiration-only protocols carried-out at a MSCT or 2nd-DSCT system.

Our study remarkably demonstrates the reduction of patient exposure without compromising image quality (cf. Figure 2) due to the technical improvements of CT systems achieved over the last years by the example of COPD patients undergoing chest imaging at three consecutive generations of CT systems. This has been achieved by various hardware and software innovations such as more efficient detectors, automated tube current modulation, reduced tube voltages, dynamic collimation, and iterative image reconstruction algorithms. According to our findings, however, the greatest effect results from spectral shaping by tin filters available with the latest generation of DSCT systems [11].

The effective dose is frequently used as a generic measure for the radiation risk associated with the exposure of patients by radiological imaging procedures. It is defined as the tissue-weighted sum of the equivalent organ doses. It has been developed by the ICRP as a dose quantity related to stochastic radiation risk, mainly cancer, and is applied to a reference person of averaged age and sex [16,25]. Therefore, this approach was only used for comparative purposes in the present study [25]. The mean effective doses for the singe-only protocols were 4.3 ± 1.5 mSv at the MSCT, 3.0 ± 1.2 mSv at the 2nd-DSCT, and 1.0 ± 0.4 mSv at the 3rd-DSCT. The effective dose for the paired inspiratory-expiratory protocol used at the 3rd-DSCT was 2.0 ± 0.8 mSv, which corresponds to the average natural radiation exposure per year in Germany.

To assess the potential effects of a radiation exposure for individual patients or a particular group of patients, it is necessary to consider organ-, sex-, and age-specific risk models, as has been done in the present study. The LAR estimates for the considered chest CT scans yield a noticeably higher cancer risk for women compared to men, mainly due to their higher risk for radiation-induced breast and lung cancer. For a representative female or male patient examined at the age of 50, the LAR for the paired inspiratory-expiratory chest scan at the 3rd-DSCT system was estimated to be 0.025% or 0.013%, respectively, which corresponds to about one hypothetical excess cancer in 4000 female or 7700 male patients. These values have to be compared to the lifetime baseline cancer risk (incidence excluding non-melanoma skin cancer) of a 50-year old woman or man in Germany of about 40 and 50%, respectively [26]. The average additional cancer risk associated with even a few chest scans is thus small for COPD patients. This risk assessment applies to patients suffering from COPD which is most prevalent in older persons. As radiation risks increase considerably with decreasing age at exposure, the presented risk assessment cannot thoughtlessly be transferred to younger patients suffering from other lung diseases.

Previous studies analyzed the radiation exposure of unenhanced chest CT scans between different CT systems [27,28,29,30]. However, these studies did not focus on a paired inspiration-expiration protocol, nor did they evaluate organ doses or cancer risks. For example, Braun et al. analyzed the radiation exposure (CTDI_vol_, DLP and size-specific dose estimate) of unenhanced chest CT scans acquired in maximal inspiration at a 2nd- and 3rd-DSCT [30]. They reported similar results compared to our study, stating the benefit of spectral shaping in thorax imaging. Gordic et al. showed that inspiratory chest scans performed at a 3rd-DSCT improve the detection of pulmonary nodules at a reduced radiation dose and better image quality compared to scans performed at a 2nd-DSCT [28].

Four aspects of our study may be critically questioned: (i) Examinations of COPD patients were performed at three different CT systems from only one manufacturer. This certainly limits the general applicability of our results. But on the other hand, it offers the possibility to better assess the relevance of specific technical components, such as the effect of spectral shaping by an optional retractable tin filter available at the high-end FORCE CT. Furthermore, similar techniques can be expected not only from other manufacturers but also for lower class devices in the near future. (ii) The inclusion of a 16-slice MSCT in the comparative evaluation of the radiation exposure to COPD patients at different CT systems. However, such scanners are still widely in use today for routine chest examinations in many facilities [31,32,33]. Furthermore, patient exposure is also substantially lower for the paired inspiratory-expiratory protocol used at the 3rd-DSCT compared to the inspiration-only protocol used at the 2nd-DSCT. (iii) The relatively small number of COPD patients examined at the 3rd-DSCT due to the different scanner usage in our clinic. However, due to the large differences in the dose levels determined for the three scanners, this does not question the basic result of our study. (iv) The use of the linear non-threshold (LNT) dose-response model for estimating stochastic radiation risks. Since experimental and radio-epidemiological studies do not provide conclusive evidence for the carcinogenicity of low levels of radiation (<about 50 mGy), there is a considerable controversy on the validity of the LNT model in the low-dose range [34,35]. Although the risks evaluated at low dose levels by the BEIR VII models is thus hypothetical, it is a prudent and precautionary approach to assume that it exists and that the underlying LNT hypothesis yields an upper bound [16,36].

In conclusion, our study demonstrates that paired inspiratory-expiratory scans can be acquired at DSCT systems of the latest generation without compromising image quality at lower radiation doses and thus less cancer risks compared to inspiration-only scans frequently performed in clinical routine at somewhat older CT systems operating at a lower technical level. This offers promising prospects for an improved diagnosis and therapy management of COPD patients as recent studies have shown, for example, that quantitative CT parameters are able to predict disease progression while lung function tests are still at normal physiological levels [37].

## Figures and Tables

**Table 1:** Protocol settings and reconstruction algorithms used at the three considered computed tomography (CT) systems.

	Emotion 16	Definition FLASH	FORCE
Tin filter	No	No	Yes
Automated tube voltage modulation	Yes	Yes	Yes
Tube voltage [kVp]	130	120	120
Reference current [mAs]	50	50	96
Pitch	0.8	0.6	1.2
Rotation time [s]	0.6	0.3	0.25
Detector collimation [mm]	16 × 1.2	128 × 0.6	192 × 0.6
Iterative reconstruction	No	Yes	Yes
Slice thickness [mm]	1.5	1.5	1.5
Size of reconstructed images	512 × 512	512 × 512	512 × 512

**Table 2:** Age, water-equivalent diameter (WED), and scan length of the female and male patients examined in inspiration at the three considered CT systems as well as the resulting effective doses.

	Emotion 16		Definition FLASH		FORCE	
	♀	♂	♀	♂	♀	♂
No. of patients	272	282	64	156	21	29
Age [years]	64 ± 17	64 ± 15	58 ± 14	59 ± 19	62 ± 14	60 ± 12
WED [mm]	285 ± 32	297 ± 28	281 ± 32	292 ± 30	309 ± 36	331 ± 27
Scan length [mm]	309 ± 21	327 ± 23	291 ± 32	303 ± 57	302 ± 32	321 ± 19
Effective doses [mSv]	4.3 ± 1.5		3.0 ± 1.2		1.0 ± 0.4	

**Table 3:** Equivalent doses (MW ± SD in mSv) for the ten most-highly exposed organs determined separately for female and male patients undergoing at the three considered CT systems either inspiration-only or paired inspiration-expiration scans of the lungs.

	Emotion 16		Definition FLASH		FORCE					
Breathing Position	Inspiration		Inspiration		Inspiration		Expiration		Paired	
	♀	♂	♀	♂	♀	♂	♀	♂	♀	♂
Liver	5.5 ± 2.0	5.6 ± 1.7	3.8 ± 1.3	3.7 ± 1.6	1.3 ± 0.6	1.4 ± 0.5	1.3 ± 0.6	1.4 ± 0.4	2.6 ± 1.2	2.8 ± 1.0
Adrenals	7.2 ± 2.6	7.4 ± 2.3	5.2 ± 1.7	5.1 ± 2.2	1.6 ± 0.8	1.8 ± 0.7	1.6 ± 0.8	1.8 ± 0.6	3.2 ± 1.5	3.6 ± 1.2
Skeleton	7.3 ± 2.6	7.6 ± 2.4	5.4 ± 1.8	5.3 ± 2.3	1.6 ± 0.8	1.9 ± 0.7	1.6 ± 0.8	1.8 ± 0.6	3.2 ± 1.5	3.7 ± 1.3
Heart	7.9 ± 2.8	8.5 ± 2.7	6.1 ± 2.0	6.1 ± 2.6	1.7 ± 0.9	2.0 ± 0.8	1.7 ± 0.9	2.0 ± 0.6	3.5 ± 1.6	4.0 ± 1.4
Breast	9.3 ± 3.3	-	7.2 ± 2.4	-	2.0 ± 1.0	-	2.0 ± 1.0	-	4.0 ± 1.9	-
Thyroid	9.6 ± 3.4	9.0 ± 2.8	4.7 ± 1.6	4.0 ± 1.7	2.0 ± 1.0	2.2 ± 0.8	2.0 ± 1.0	2.1 ± 0.7	4.0 ± 1.9	4.3 ± 1.5
Upper Airways	9.6 ± 3.4	9.0 ± 2.8	4.7 ± 1.6	4.0 ± 1.7	2.0 ± 1.0	2.0 ± 0.8	2.0 ± 1.0	2.1 ± 0.7	4.0 ± 1.9	4.3 ± 1.5
Esophagus	9.7 ± 3.5	9.7 ± 3.0	7.4 ± 2.5	7.0 ± 3.0	2.1 ± 1.0	2.3 ± 0.9	2.1 ± 1.0	2.3 ± 0.7	4.2 ± 2.0	4.6 ± 1.6
Lungs	9.7 ± 3.5	10.1 ± 3.2	7.4 ± 2.5	7.2 ± 3.0	2.2 ± 1.1	2.4 ± 0.9	2.2 ± 1.1	2.4 ± 0.8	4.3 ± 2.0	4.8 ± 1.7
Thymus	9.7 ± 3.5	9.8 ± 3.0	7.4 ± 2.5	7.0 ± 3.0	2.1 ± 1.0	2.3 ± 0.9	2.1 ± 1.0	2.3 ± 0.7	4.2 ± 2.0	4.6 ± 1.6
